# Draft genome sequence of emerging *Salmonella enterica* serovar Kentucky ST198 isolated from broiler litter at an integrated poultry-aquaculture marketplace in Bangladesh

**DOI:** 10.1128/mra.00514-26

**Published:** 2026-10-13

**Authors:** Bibi Amena, Mohimanul Islam, Eaftekhar Ahmed Rana, Md. Moksedul Momin, Gous Miah, Gazi Md. Noor Uddin, Tahmina Bilkis

**Affiliations:** 1 Department of Microbiology and Veterinary Public Health, Faculty of Veterinary Medicine, Chattogram Veterinary and Animal Sciences University (CVASU) 130057, Chattogram, Bangladesh; 2 Department of Genetics and Animal Breeding, Faculty of Veterinary Medicine, Chattogram Veterinary and Animal Sciences University (CVASU) 130057, Chattogram, Bangladesh; 3 Department of Nutrition and Food Engineering, Daffodil International University, Daffodil Smart City (DSC) 130058, Savar, Dhaka, Bangladesh; University of Pittsburgh School of Medicine, Pittsburgh, Pennsylvania, USA

**Keywords:** foodborne pathogen, *Salmonella* Kentucky, whole-genome sequencing, zoonoses

## Abstract

Integrated live poultry-aquaculture marketplaces represent high-risk One Health interfaces where environmental circulation of zoonotic *Salmonella* poses a threat to public health. Using Illumina NovaSeq, this study delineates genomic features of a *Salmonella* Kentucky ST198 strain, providing insight into a globally disseminated lineage and a resource for future surveillance and epidemiological investigations.

## ANNOUNCEMENT

*Salmonella enterica* serovar Kentucky ST198 has emerged as a high-risk multidrug-resistant lineage and a significant global public health concern (1). While genomic surveillance of *Salmonella* Kentucky in poultry and fish market environments in Bangladesh has recently begun to emerge (2–4), data regarding its circulation within mixed-commodity ecosystems such as integrated poultry and fish markets remain fundamentally scarce. Understanding these interfaces is crucial for assessing potential cross-contamination and clonal transmission between land- and water-based animals.

In February 2024, a surveillance study was conducted at an integrated live bird and fish market in Chattogram city (22.357612° N, 91.807461° E). Samples included pooled floor litter from chicken stalls and shrimp samples from adjacent chicken stalls. The samples were pre-enriched in buffered peptone water at a 1:10 ratio and incubated overnight at 37°C. The suspensions were streaked on Brilliant Green Agar and incubated for 24 h at 37°C, followed by subculturing characteristic colonies onto Salmonella-Shigella agar for another 24 h at 37°C. Two isolates were successfully recovered from broiler floor litter, while no *Salmonella* was isolated from shrimp samples. The isolates were confirmed through several biochemical tests and genus-level PCR using ST11–ST15 primers (5). For whole-genome sequencing, one isolate was re-cultured for 24 h on 5% bovine blood agar at 37°C. Genomic DNA was extracted from a single colony using the FavorPrep Bacterial Genomic DNA Extraction Kit (Favorgen, Taiwan). The quality and concentration of the genomic DNA were checked using a NanoDrop spectrophotometer (Thermo Fisher Scientific, USA). Genome sequencing was performed on the Illumina NovaSeq X Plus platform at Novogene (China), generating 2 × 150 bp paired-end reads. Libraries were prepared using the Illumina DNA Prep kit (Illumina, San Diego, CA, USA) according to the manufacturer’s instructions. Bioinformatic analyses were conducted via the Galaxy platform v25.0 (6). The quality of the raw reads was checked using FastQC v0.12.1 (7). Trimmomatic v0.39 (8) was used to trim low-quality bases and adapter sequences. *De novo* genome assembly was performed using Shovill v1.1.0 (9), utilizing SPAdes v4.2.0 (10). The quality of the assembled genome was checked using QUAST v5.3.0 (11). Kraken2 v2.17.1 (12) was used to confirm the taxonomic identity. Antimicrobial resistance genes with chromosomal point mutations, plasmid replicons, and *Salmonella* pathogenicity islands (SPIs) were identified using ResFinder v4.7.2 (13–15), PlasmidFinder v2.0.1 (15, 16), and SPIFinder v2.0 (17), respectively. The strain was predicted to be a human pathogen using PathogenFinder v1.1 (15, 18). Default parameters were used for all software except where otherwise noted. The genome was annotated using the NCBI Prokaryotic Genome Annotation Pipeline (PGAP) v6.10 (19). The features of the draft genome are presented in Table 1. Multi-locus sequence typing based on seven housekeeping genes (*aroC*, *dnaN*, *hemD*, *hisD*, *purE*, *sucA*, and *thrA*) via the PubMLST database (20) identified the isolate as ST198 within clonal complex (CC) 56.

**TABLE 1:** Genomic features of the *S. enterica* serovar Kentucky strain Sal-2

Strain ID	Sal-2
Species	*S. enterica* serovar Kentucky
Sample	Floor litter of broiler chickens
BioSample accession number	SAMN50545291
SRA accession number	SRR34936019
Genome assembly accession number	GCA_053570095.1
GenBank accession number	JBSEEG000000000
Number of raw reads	7,711,269
Sequencing depth (×)	449
Number of reads	7,665,934
Genome size (bp)	4,894,650
Number of contigs	50
Largest contig (bp)	2,003,623
Contig N50 (bp)	773,722
Contig L50	2
GC content (%)	52.12
Genes (total), PGAP	4,809
CDSs (total)	4,694
rRNAs	8, 4, 9 (5S, 16S, 23S)
tRNAs	80
CRISPR arrays	2
Clonal complex (CC)	56
Sequence type (ST)	198
Antimicrobial resistance genes (ResFinder)	*aac(6')-Iaa, blaTEM-1B*
Chromosomal point mutations (PointFinder)	*gyrA* p.S83F, *gyrA* p.D87Y, *parC* p.T57S, *parC* p.S80I
Plasmids (PlasmidFinder)	IncI1-I(Alpha), IncQ1
*Salmonella* pathogenicity islands (SPIs) (SPIFinder)	C63PI, SPI-1, SPI-2, SPI-3, SPI-4, SPI-5, SPI-9
Probability of being a human pathogen (PathogenFinder)	0.934

The identification of a member of the globally disseminated ST198 lineage in Bangladeshi live poultry-aquaculture market environments demonstrates the critical need for integrated genomic surveillance of *Salmonella* across the terrestrial and aquatic food-animal systems. These data are expected to serve as a genomic resource for future epidemiological studies in the region.

## Data Availability

The whole-genome sequence data have been deposited at NCBI/GenBank under the BioProject accession number PRJNA1303988, with metadata provided in Table 1.
